# Clinical Factors and Social Determinants of Health Predict Elder Mistreatment at Two Years Among Older Adults: A Preliminary Prediction Model

**DOI:** 10.1177/00469580251375869

**Published:** 2025-10-16

**Authors:** Monique R. Pappadis, Karen E. Schlag, Jordan Westra, Leila Wood, Rebecca Czyz, Yong-Fang Kuo, Mukaila A. Raji, Jeff R. Temple, Charles P. Mouton

**Affiliations:** 1 The University of Texas Medical Branch (UTMB Health) at Galveston, TX, USA; 2 UT Health Houston, TX, USA

**Keywords:** aged, elder abuse, medicare, risk assessment, social determinants of health

## Abstract

Low rates of screening for elder mistreatment preclude early detection and harm reduction. No validated tools currently exist to identify elder mistreatment with high sensitivity. We developed, internally validated, and tested a preliminary prediction model to improve elder mistreatment screening of older adults. This retrospective observational study used 20% national Medicare Fee-for-Service claims data for the logistic regression and machine learning approaches (random forest, gradient boosted decision tree, and multilayer perceptron classifier). We included beneficiaries aged 66 and older with no elder mistreatment diagnosis from January 1, 2015 through December 31, 2016; continuously enrolled in Medicare Parts A, B, and D with no HMO coverage; and followed through 2018 (n = 2 261 166). The primary outcome was elder mistreatment diagnosis between January 1, 2017 and December 31, 2018. Predictors included demographic characteristics, comorbidities, health symptoms, and medical and social factors (ie, social determinants of health). Data analyzed were from June 23, 2022 to November 4, 2024. The sample was diverse (eg, 60.9% female, 15.2% racial and ethnic minorities, 14.6% Medicaid dual eligible, 8.9% disability prior to age 65, 72.6% pre-frail, 11.4% dementia, 64.6% hypertension). Overall, elder mistreatment diagnosis was low at 0.2%. An elder mistreatment prediction model with the best model performance was identified (c-statistic: 0.7253; 95% Confidence Interval [95% CI]: 0.712-0.738; sensitivity = 0.801, specificity = 0.467, positive predictive value = 0.002, negative predictive value = 0.999). Primary patient/caregiver support challenges (odds ratio [OR], 3.55; 95% CI: 2.36-5.34), housing and income problems (OR, 2.70; 95% CI: 1.59-4.61), learning disabilities (OR, 2.35; 95% CI: 1.15-4.78), and being tested for sexually transmitted infections (OR, 2.25; 95% CI: 1.44-3.53) were the top 4 predictors associated with increased risk of elder mistreatment diagnosis. Our preliminary elder mistreatment prediction model may guide the development of a risk assessment tool to facilitate clinician screening practices and implementation of interventions to better protect older adults from mistreatment.

## Introduction

One in every 6 older adults experiences elder mistreatment,^1^ defined as an intended action or non-action by a trusted individual leading to harm or risk of harm,^2^ such as abuse (physical, emotional, sexual, and financial) and neglect. Elder mistreatment has been consistently linked to adverse health outcomes such as metabolic syndrome,^3^ hospitalization and readmission,^4^ mortality,^5^ and increased medical costs.^6^ Risk factors for elder mistreatment include patient functional and cognitive impairment,^7^ chronic health conditions,^8^ and social determinants of health (SDoH) such as poverty, marital problems, and lack of social support.^9,10^

Despite considerable research and intervention efforts to identify risk factors for and prevent elder mistreatment,^7,10^ abuse of older adults continues to go mostly undetected.^11^ This reality is, in part, due to a lack of validated risk assessment tools for use in diverse settings. While validated screening tools exist to identify signs of elder mistreatment,^11,12^ development of these instruments has typically drawn from homogenous populations^13^ and relied on varied psychometric properties for evaluation.^14^ Additionally, screening instruments used to assess EM across diverse populations have not fully considered social or cultural factors that may contribute to abuse characteristics, nor have they adequately addressed concerns specific to vulnerable older adults, such as those with cognitive impairment.^12^ Moreover, the suitability of different screeners, which generally rely on older adult or family caregiver self-reports to identify abuse characteristics, is dependent on the setting where administered.^11^

While important cross-sectional research has identified some risk and protective factors of elder mistreatment, mixed or limited evidence exists on sociodemographic characteristics and SDoH as risk factors.^13^ Using Medicare data, 1 study identified patient conditions and characteristics associated with formal elder mistreatment diagnoses, such as dementia and other psychiatric diagnoses as well as cardiovascular disease and other physically disabling diseases.^15^ There is also a conspicuous lack of longitudinal studies with diverse populations.^13,16^ To date, only 1 published study has used an advanced analytical approach to identify correlates of elder mistreatment.^17^ Development of an elder mistreatment prediction model that incorporates social and economic determinants of health and frailty status could meet a gap to improve the accuracy of identifying individuals who may be at greatest risk of experiencing elder mistreatment in primary and ambulatory care settings.^18^ In consideration of these limitations, a comprehensive risk model that accounts for individual-level sociodemographic characteristics, psychosocial factors, and physical and cognitive functioning is needed to aid elder mistreatment mitigation and prevention efforts.^19^ This is important given that no single recommended screening tool, and no empirical evidence exist on the screening of older adults at risk to predict future elder mistreatment.^11^

Prediction models using advanced machine learning techniques and drawing from health insurance claims data have been used to improve detection accuracy of health risks for older adults, including mortality,^20^ long-term care needs,^21^ dementia,^22^ Parkinson’s disease,^23^ and falls.^24^ In addition, prediction models have also identified low incident and underdiagnosed conditions, such as mild cognitive impairment.^25^ Artificial intelligence/machine learning approaches may provide greater accuracy with identifying patients at high risk of incident diagnoses^26^ and poorer outcomes such as mortality.^27^ These approaches may be implemented using existing electronic health records or used to develop health risk assessments.^28^

In consideration of the limitations of prior work, we believe that using advanced statistical approaches to predict elder mistreatment can guide clinicians in identifying early signs of elder mistreatment and support screening practices; help identify older adults at greatest risk for elder mistreatment-related adverse outcomes who may need a more comprehensive clinical evaluation; and guide the referral to needed local and state resources.^29^ To meet a need and identify EM elder mistreatment risk and protective factors with greater precision to guide healthcare professionals in identifying high-risk individuals and initiating referrals for needed support, our research objective was to develop, test, and internally validate a preliminary elder mistreatment risk prediction model using national Medicare data. In doing so, we addressed the following research question: What medical, social, and environmental factors are the most predictive of receiving an elder mistreatment diagnosis among Medicare beneficiaries aged 66 and older?

## Methods

### Data Source

We used a 20% national sample of Medicare Fee-for-Service beneficiaries. The Medicare database included information on beneficiary demographics from the Master Beneficiary Summary File (MBSF); diagnosis and procedure information from the carrier, outpatient, and the Medicare Provider Analysis and Review (MedPAR) files; chronic conditions from the MBSF Chronic Conditions Segment; and prescription drug information from the Part D event files.

### Study Cohort

We selected living Medicare Fee-for-Service beneficiaries aged 66 and older with Medicare parts A, B, and D coverage and no HMO in 2015 and 2016 through the end of 2018. We excluded those with a diagnosis of elder mistreatment in 2015 or 2016. A total of 2 216 166 Medicare beneficiaries were included (Table S1). The first author’s Institutional Review Board approved the study with a waiver of informed consent and HIPAA authorization. A data use agreement was obtained with the Center for Medicare and Medicaid Services (CMS). We followed the latest Transparent Reporting of a Multivariable Prediction Model for Individual Prognosis or Diagnosis and Artificial Intelligence (TRIPOD + AI) reporting guidelines.^30^

### Elder Mistreatment Diagnosis

Consistent with a prior study^4^ and the Office of Inspector General’s report,^31^ we used International Classification of Diseases, Tenth Revision (ICD-10) codes to identify elder mistreatment (T74.0-T74.3, T74.5, T74.9, T76.0-T763, T76.5, T76.9, Z65.8, Z65.9). Elder mistreatment diagnoses during an inpatient hospital and skilled nursing facility stay were identified using the MedPAR file. Elder mistreatment diagnoses identified by non-institutional healthcare providers during office or outpatient visits were found in the Carrier file, while the Outpatient file included diagnoses from institutional outpatient providers.

### Predictors of Elder Mistreatment Diagnosis

We classified predictors into 4 categories: demographics [age (66-70, 71-75, 76-80, 81-85, 86+ years), sex (male, female), race (White, Black, Asian, Hispanic, American Indian/Alaska Native, Other, Unknown), Medicaid dual eligibility (yes, no), and original Medicare entitlement reason (age 65+, disability and end-stage renal disease – ESRD)], comorbidities, symptoms, and other social/medical factors (Table S2). The MBSF included demographics and the MBSF Chronic Conditions Segment included comorbidities. Prescription drug information was obtained from the Part D event files. Symptoms/signs were created from medical claims and also included injury history and the Medicare claims frailty score (0-1).^32^ Other social/medical factors were derived from medical and procedure claims as well as SDoH.

### Statistical Analysis

The study involved cohort analysis and variable selection, dividing the cohort into training (50%), validation (25%), and internal test (25%) subsets. Variables with low frequency (n < 600) or collinearity were excluded. We calculated the area under the curve (AUC) and created a receiver operating characteristic (ROC) curve for the best model using the training dataset. We ran 4 logistic regression analyses predicting elder mistreatment diagnosis, sequentially adding demographics, comorbidities, symptoms, and social/medical factors, calculating odds ratios (OR) and 95% confidence intervals (95% CI) for each variable.

Using the internal test set, we calculated predicted probabilities of elder mistreatment diagnosis and created a calibration plot based on those probabilities. We evaluated sensitivity, specificity, positive predictive value (PPV), and negative predictive value (NPV) at various cutoff percentiles of predicted probability of EM diagnosis. We employed artificial intelligence/machine learning methods, including random forest (RF), gradient-boosted tree classification (GBDT), and multilayer perceptron (MLP) classifiers. These models used the training, validation, and testing sets to create the best prediction model for each method. We used cross-validation to find the best combination of options and optimize model parameters (RF: change number of trees and depth, and impurity type; GDBT: tree depth and step size; MLP classifier: hidden layer size), and various metrics (AUC, percent agreement, recall, purity, and *f*-measure). ROC curves were created for each method.

Analyses utilized SAS Enterprise Guide 7.15 (SAS Institute Inc., Cary, NC) and R within the Databricks software system at CMS Virtual Research Data Center.

## Results

### Study Sample Characteristics

We included 2 261 166 Medicare beneficiaries aged 66 and older (60.9% female, 84.8% non-Hispanic White [NHW], 14.6% Medicaid dual eligible, 8.9% with a disability prior to age 65 [1.6% with ESRD], 56% ≤75 years; Table 1). The overall rate of elder mistreatment diagnosis for the total sample was 0.2% (N = 4648), with 93.77% diagnosed in an outpatient setting, 4.9% in inpatient, and 1.34% diagnosed in both settings. Commonly reported symptoms included general symptoms, gait abnormalities, skin conditions, history of falling, and fractures. Most older adults were pre-frail (72.6%). A variety of cardiovascular comorbidities and some SDoH were identified (See Table S3). The baseline characteristics of the training, validation, and testing cohorts are provided (See Table S4). The 5 most common comorbidities were hypertension (64.6%), hyperlipidemia (54.1%), rheumatoid arthritis/osteoarthritis (37.4%), ischemic heart disease (29.3%), and diabetes (28.5%). Commonly reported symptoms included general symptoms (41.1%, such as fever, headache, edema, and fatigue), gait abnormalities (16.8%), skin conditions (12.5%, such as skin sensations, swelling, changes), history of falling (6.0%), and fractures (2.4%). Frailty scores were non-frail, 8.9%; pre-frail, 72.6%; mildly frail, 16.1%; moderately frail, 2.4%; and severely frail, 0.1%. Overall, SDoH were not frequently reported in the claims data, with marital problems (2.8%) reported most often.

**Table 1.** Demographic Characteristics and Symptoms of the Total Cohort.

	Total	
Cohort characteristics	N	%
Total	2 261 166	100
Elder mistreatment diagnosis		
No	2 256 518	99.8
Yes	4648	0.2
Sex		
Male	883 090	39.1
Female	1 378 076	60.9
Race		
Unknown	28 126	1.2
White	1 916 661	84.8
Black	129 078	5.7
Other	16 400	0.7
Asian/Pacific Islander	62 666	2.8
Hispanic	100 399	4.4
American Indian/Alaska Native	7836	0.3
Medicaid dual eligible		
No	1 931 292	85.4
Yes	329 874	14.6
Original entitlement		
Disability	202 008	8.9
Age 65+	2 059 158	91.1
Age group		
66-70	633 017	28.0
71-75	636 651	28.2
76-80	452 872	20.0
81-85	289 785	12.8
86+	248 841	11.0
General symptoms	929 114	41.1
Skin symptoms	282 973	12.5
Gait abnormalities	372 689	16.5
History of fracture	53 644	2.4
History of fall	135 976	6.0
Frailty score	0.16	0.05
Non-frail (≤0.1)	200 352	8.9
Pre-frail (0.1-0.2)	1 641 511	72.6
Mild frailty (0.2-0.3)	363 149	16.1
Moderate frailty (0.3-0.4)	53 640	2.4
Severe frailty (≥0.4)	2514	0.1

### Risk and Protective Factors of Elder Mistreatment

As shown in Table 2, being diagnosed due to concern related to social support or social environment was the greatest predictor of a diagnosis of elder mistreatment within a 2-year follow-up period (OR = 3.55; 95% CI: 2.36, 5.34), followed by housing and income problems (OR = 2.70; 95% CI: 1.59, 4.61). Other SDoH (eg, Medicaid dual and disability eligibility, Black race, etc.) were strongly associated with elder mistreatment diagnosis during follow-up. Having a learning disability, sexually transmitted infection (STI) testing, certain chronic conditions (eg, alcohol use disorders, viral hepatitis, lung cancer, liver disease, etc.), and mental health conditions (ie, personality disorders, anxiety, bipolar disorder, and depressive disorders) were associated with elder mistreatment diagnosis at 2 years. Several physical signs and symptoms were also associated with an increased risk of elder mistreatment diagnosis. A decreased risk of elder mistreatment diagnosis was associated with hip fracture, depression screening, cataracts, hyperlipidemia, and hypertension.

**Table 2.** Significant Predictors of Elder Mistreatment (EM) at 2 years using Logistic Regression.

Associated with **increased** odds of EM at 2 years		Associated with **decreased** odds of EM at 2 years	
Factor	OR (95% CI)	Factor	OR (95% CI)
Primary support challenges	3.55 (2.36, 5.34)	Hip fracture	0.43 (0.22, 0.82)
Housing and income problems	2.70 (1.59, 4.61)	Depression screening	0.72 (0.53, 0.97)
Learning disabilities	2.35 (1.15, 4.78)	Cataracts	0.80 (0.70, 0.91)
Sexually transmitted infections testing	2.25 (1.44, 3.53)	Hyperlipidemia	0.80 (0.72, 0.90)
Cancer lung	1.93 (1.34, 2.78)	Hypertension	0.84 (0.74, 0.96)
Social environmental problems	1.91 (1.09, 3.35)		
Alcohol use disorders	1.64 (1.26, 2.14)		
Marital problem	1.59 (1.25, 2.01)		
Personality disorders	1.55 (1.16, 2.08)		
Medicaid dual eligibility	1.54 (1.35, 1.75)		
Liver disease	1.51 (1.24, 1.84)		
Leukemia/lymphoma	1.47 (1.08, 2.01)		
Viral hepatitis	1.47 (1.01, 2.14)		
Disability (vs medicare age eligible)	1.39 (1.20, 1.60)		
General symptoms	1.39 (1.23, 1.57)		
Anxiety	1.36 (1.19, 1.55)		
Ulcers	1.36 (1.11, 1.66)		
Black race	1.32 (1.09, 1.58)		
Bipolar disorders	1.31 (1.03, 1.66)		
Chronic kidney disease	1.29 (1.14, 1.47)		
Abnormal weight loss	1.26 (1.03, 1.55)		
Depressive disorders	1.23 (1.07, 1.42)		
Skin symptoms	1.19 (1.04, 1.35)		
Frailty score			
Per 0.01 increase	1.04 (1.03, 1.06)		
Per 0.05 increase	1.22 (1.13, 1.32)		
Per 0.10 increase	1.50 (1.28, 1.75		

*Note.* EM = elder mistreatment.

### Elder Mistreatment Risk Model Validation

We identified an elder mistreatment prediction model with the best overall performance including demographics, comorbidities, symptoms, and social/medical factors (see Table S5 and Figure S1). The predicted and observed probabilities of elder mistreatment diagnosis calibration plots are provided in Table S6 and Figure S2, respectively. We also examined various cutoff points (Table S7).

Two-way and three-way interactions were tested by sex, race, and Medicaid dual eligibility (Table S8). The model performed better for ethnic minority and male older adults (No Medicaid: c-statistic = 0.85; 95% CI: 0.78-0.92, Medicaid: c-statistic = 0.85; 95% CI: 0.80-0.89) than for NHW and female older adults (No Medicaid: c-statistic = 0.70; 95% CI: 0.68-0.72, Medicaid: c-statistic = 0.73; 95% CI: 0.69-0.77). Findings were mixed for those who were Medicaid dual eligible, where the model for females with Medicaid did not perform as well as those with Medicaid dual eligibility as did the model for males.

Given the differences by sex and race, we stratified our findings by sex (Table S9) and race (Table S10). Muscular dystrophy (OR = 6.67; 95% CI: 1.57-28.30) and housing and income problems (OR = 3.84; 95% CI: 1.86, 7.92) were the most predictive factors for elder mistreatment among males. For females, primary support issues (OR = 3.63; 95% CI: 2.32-5.38) and testing for STIs (OR = 2.63; 95% CI: 1.62-4.29) were most predictive of elder mistreatment. Among NHW older adults, primary support issues (OR = 3.40; 95% CI: 2.15-5.36) and being screened for HIV (OR = 3.30; 95% CI: 1.21-8.96) were strongly associated with elder mistreatment diagnosis. For other racial groups, housing and income problems (OR = 5.01; 95% CI: 2.03-12.33) and testing for STIs (OR = 4.38; 95% CI: 2.40-8.00) were most associated with elder mistreatment diagnosis.

### Artificial Intelligence/Machine Learning Models

Cross validation was done by splitting the dataset into training, validation, and testing sets and tested a variety of combinations of parameters (eg, number of trees, depth, impurity type, step size, and hidden layer size). Then, we identified the best model for each method (ie, RF, GBDT, and MLP classifier) using various metrics (AUC, percent agreement, recall, purity, and *F*-measure). We then determined variable importance for the RF and GBDT models (Table S11) and found that frailty, race, CKD, abnormal weight loss, and age were consistently among the top 10 predictors of elder mistreatment diagnosis for both methods. Other important factors include urinary tract infection diagnosis for the RF model and hypertension for the GBDT model.

### Elder Mistreatment Model Comparisons

**Figure 1.**
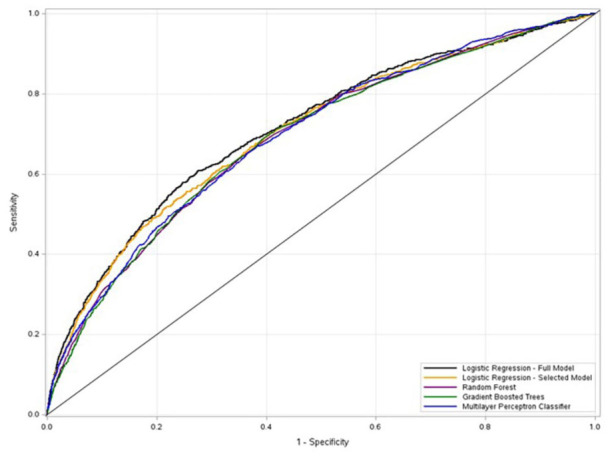
Receiver operating characteristic curve plots for logistic regression and artificial intelligence/machine learning models.

Using the cross-validation metrics, the full LR model performed better than the artificial intelligence/machine learning models (AUC = 0.7253; sensitivity = 0.8007; specificity = 0.4672; % agreement = 46.77%; See Table S12 and Figure 1). The RF model had the greatest sensitivity (0.8119), GBDT had the best purity (GINI Impurity = 0.4973), and although extremely low and suggesting poor precision, the MLP classifier model had the best *F*-measure (0.0044). All models were optimized to have high recall or sensitivity (minimize false negatives), resulting in lower precision (increasing false positives). The ROC curve cut points for each model are included in Table S13.

## Discussion

This is the first retrospective observational study to build, internally validate, and test a preliminary elder mistreatment prediction model with national data with the greatest sensitivity to correctly identify those diagnosed with elder mistreatment at 2 years. Our elder mistreatment prediction model performed better for underrepresented ethnic minority and male older adults. This finding may represent a greater willingness of clinicians to list a diagnosis of elder mistreatment in these populations, or it may represent an increased severity of EM markers in these populations, thus increasing the likelihood of identification of elder mistreatment. In addition, mixed results were identified for those with Medicaid. Further evaluation of subgroup differences will guide clinicians in identifying risk factors disproportionally affecting certain older adults.

We identified several risk and protective factors associated with an elder mistreatment diagnosis within 2 years. Risk factors include disability, SDoH, screening tests, and health-related conditions and symptoms. Factors associated with decreased risk for elder mistreatment included hip fracture diagnosis, depression screening, cataracts, hyperlipidemia, and hypertension. We identified important and consistent predictors of elder mistreatment, such as frailty, race, CKD, abnormal weight loss, and age.

Consistent with prior studies, elder mistreatment screening rates were low.^33-35^ We found an overall elder mistreatment diagnosis rate of 0.2%, which translates to roughly 1 out of 500 Medicare patients seen in an outpatient primary care setting. CMS has an elder mistreatment screening quality metric for clinicians to document screening and follow up (if positive) for older Medicare beneficiaries at least once per year. Despite this established metric, elder mistreatment screening rates remain low.

Although previous studies identified some of our identified risk factors,^10,13^ their cohorts may differ from US Medicare beneficiaries. Physical signs and symptoms are commonly reported as obvious signs of potential elder abuse or neglect.^36^ However, the non-physical signs of abuse or neglect are more challenging to identify, because they can be subtle or identified only when assessing the social interactions between the potential perpetrator and the older adult. In addition, signs and symptoms of elder mistreatment are often difficult to distinguish from normal aging, as elder mistreatment is complex. This study identified unique predictors associated with elder mistreatment diagnosis not found in the literature, such as diagnosis of lung cancer, liver disease, leukemia/lymphoma, viral hepatitis, and CKD. These conditions are often associated with older patients’ declining health and frailty, which can significantly impact a family caregiver’s health, each of which are known risk factors for elder mistreatment.^10^ Screening for STI was also associated with increased risk of elder mistreatment, which may be due to suspected sexual abuse.^37^

Several factors associated with decreased risk were identified, such as having a hip fracture, cataracts, hyperlipidemia, hypertension, and depression screening. It is possible that these may serve as proxies for better engagement, access to care and preventive services, caregiver supports, and chronic care management, which may provide opportunities to protect older adults from harm. Cardiovascular-related conditions and orthopedic injuries are often monitored by clinicians because of the potential for adverse outcomes, such as heart attack, stroke, or deep vein thrombosis. Routine depression screening is also recommended for older adults, and depression is a known elder mistreatment risk factor.^10^ Our elder mistreatment prediction model may be used to complement existing elder mistreatment screening practices, guide clinicians to monitor patients’ risk over time, or help build a screening tool to support older adult patients and their families.

### Limitations

There are some limitations worth noting. First, we excluded individuals with HMO coverage, which now makes up more than half (54%) of the eligible US Medicare beneficiaries.^38^ Medicare Advantage (MA) is associated with greater use of preventative service and fewer hospital and emergency department visits compared to Medicare Fee-for-Service.^39^ However, elder mistreatment screening is also low in MA beneficiaries.^40^ Whether elder mistreatment risk factors vary between these 2 types of beneficiaries is unknown. Second, low ICD-10 diagnosis codes usage and coding errors may impact low reporting of elder mistreatment. Our elder mistreatment diagnosis prevalence is similar to rates identified in emergency departments (0.01%-0.03%),^41^ where underreporting occurs. Clinicians often report lack of formal training in recognizing elder mistreatment, fear of affecting the patient-provider relationship, and challenges with differentiating intentional from unintentional trauma.^41,42^ Combining administrative data with Adult Protective Services report data may improve the predictive risk model’s performance. Third, we optimized our model to minimize false negatives, which in turn reduced the precision of our model. Using a model with high recall and low precision is important when using the model to identify risk factors that would prompt clinicians for further evaluation and screening for actual abuse, neglect, or exploitation. Fourth, although we did not include an a priori formal power analysis of the existing Medicare Fee-for-Service data, the narrow confidence intervals suggest greater precision with estimating the uncommon diagnosis of elder mistreatment. Five, we included older adults from both institutionalized and community-based settings. Future work should consider developing and testing elder mistreatment risk models for other care settings.

### Implications for Research, Policy, and Practice

These findings and the development of this risk model have significant implications for future research and practice. First, these models can assist health professionals in clinical care settings to heighten their suspicion of elder mistreatment. One way to improve early identification of elder mistreatment is for health system to consider incorporation of the elder mistreatment prediction model into electronic health records – with alerts to clinicians regarding screening for early elder mistreatment detection. Such incorporation can be considered by policy makers as part of the Centers for Medicare & Medicaid Services-mandated Age Friendly Hospital (AFH) Measure for US health system hospitals who are participating in the Hospital Inpatient Quality Reporting (IQR) Program.^43,44^ The elder mistreatment screening tool is especially relevant to the ‘Social Vulnerability’ domain of the 5 AFH domains.^43,44^ In addition, protective factors can be considered as health providers consider their development of safety plans for individuals with possible elder mistreatment risk. This model has potential uses in multiple care settings including inpatient, emergency departments, and ambulatory settings. Future research will need to identify how the various factors change over time and influence the risk of elder mistreatment. Future studies should also consider how influences from different risk factors vary by sociodemographic factors such as sex or race. Understanding the progress of the factors in the model may present unique opportunities for intervention.

## Conclusions

Our preliminary elder mistreatment risk model successfully predicted elder mistreatment diagnosis among older Medicare Fee-for-Service beneficiaries. Risk factors included SDoH, cognitive impairments, disability, screening tests, chronic conditions, and physical signs and symptoms. Factors associated with decreased risk of elder mistreatment diagnosis included cardiovascular-related conditions and orthopedic injuries requiring continued health monitoring, as well as preventive screening for depression. These factors may guide the development of an elder mistreatment risk assessment and intervention tool to better protect older adults from mistreatment.

## Supplemental Material

sj-docx-1-inq-10.1177_00469580251375869 – Supplemental material for Clinical Factors and Social Determinants of Health Predict Elder Mistreatment at Two Years Among Older Adults: A Preliminary Prediction Model Supplemental material, sj-docx-1-inq-10.1177_00469580251375869 for Clinical Factors and Social Determinants of Health Predict Elder Mistreatment at Two Years Among Older Adults: A Preliminary Prediction Model by Monique R. Pappadis, Karen E. Schlag, Jordan Westra, Leila Wood, Rebecca Czyz, Yong-Fang Kuo, Mukaila A. Raji, Jeff R. Temple and Charles P. Mouton in INQUIRY: The Journal of Health Care Organization, Provision, and Financing
