# Supplementary material for: Clinical Factors and Social Determinants of Health Predict Elder Mistreatment at Two Years Among Older Adults: A Preliminary Prediction Model
Source: Inquiry. 2025 Oct 16;62:00469580251375869. doi: 10.1177/00469580251375869 (PMC12536136; doi:10.1177/00469580251375869)
Supplement: sj-docx-1-inq-10.1177_00469580251375869 – Supplemental material for Clinical Factors and Social Determinants of Health Predict Elder Mistreatment at Two Years Among Older Adults: A Preliminary Prediction Model [file sj-docx-1-inq-10.1177_00469580251375869.docx]

**Supplementary Table S1:** Cohort Derivation

| **Step** | **Description** | **Sample** |
| --- | --- | --- |
| 0 | All Medicare Beneficiaries in 2015-01-01 through 2016-12-31 | 4,874,475 |
| 1 | Patients aged 66 years old or older | 3,732,097 |
| 2 | Medicare Parts A/B/D eligible with no HMO in 2015 to 2016 | 2,924,933 |
| 3 | Alive at the end of 2016 | 2,910,579 |
| 4 | Medicare eligible through 2018 | 2,346,804 |
| 5 | No elder mistreatment Diagnosis in 2015-2015 | 2,261,166 |

**Supplementary Table S2:** Demographic, Comorbidity, Symptoms, and Social and Medical Factors as Predictors of Elder Mistreatment Diagnosis

| **Predictors** | **Specific Variables Included** |
| --- | --- |
| **Demographics** | Sex (male, female), race (White, Black, Asian, Hispanic, American Indian/Alaska Native, Other, Unknown), Medicaid dual eligibility (yes/no), original Medicare entitlement reason (age 65+, disability and End Stage Renal Disease), and age (66-70, 71-75, 76-80, 81-85, 86+ years) |
|  |  |
| **Comorbidities** |  |
| Cardiovascular Conditions | Acute myocardial infarction, atrial fibrillation, heart failure, hypertension, ischemic heart disease, peripheral vascular disease |
| Cancer | Breast cancer, colorectal cancer, endometrial cancer, leukemia and lymphomas, lung cancer, prostate cancer |
| Cognitive, Mental, and Behavioral Health  Conditions | Attention-Deficit/Hyperactivity Disorder and other conduct disorders, alcohol use disorders, Alzheimer's disease and related disorders or senile dementia, anxiety disorders, autism, bipolar disorder, depression, drug use disorder, intellectual disabilities and related conditions, learning disabilities, major depressive affective disorder, schizophrenia and other psychotic disorders, other developmental delays, personality disorders, post-traumatic stress disorder, tobacco use disorders, overarching opioid use disorder |
| Digestive Conditions | Liver disease, cirrhosis and other liver conditions (excluding hepatitis) |
| Endocrine, Nutritional, and Renal Conditions | Acquired hypothyroidism, anemia, chronic kidney disease, cystic fibrosis and other metabolic developmental disorders, diabetes, hyperlipidemia, obesity |
| Genitourinary Conditions | Benign prostatic hyperplasia, |
| Infectious Diseases | Human Immunodeficiency Virus and/or Acquired Immunodeficiency Syndrome, viral hepatitis (general) |
| Musculoskeletal and Joint Conditions | Chronic pain and fatigue, fibromyalgia, Hip/pelvic fracture, mobility impairments, osteoporosis, rheumatoid arthritis/osteoarthritis, |
| Neurologic Conditions | Cerebral palsy, epilepsy, stroke/transient ischemic attack, traumatic brain injury and nonpsychotic mental disorders due to brain damage, migraine and other chronic headache, multiple sclerosis and transverse myelitis, muscular dystrophy, spina bifida and other congenital anomalies of the nervous system, spinal cord injury, |
| Respiratory Conditions | Asthma, chronic obstructive pulmonary disease, |
| Ophthalmic and Hearing  Conditions | Blindness and visual impairment, cataract, deafness and hearing impairment, glaucoma, |
| Skin Conditions | Pressure ulcers and chronic ulcers |
|  |  |
| **Symptoms/Signs** | Abnormal weight loss (R63.4), age-related physical disability (R54), cachexia (R64), convulsions (R56), demoralization and apathy (R45.3), edema (R60), enlarged lymph nodes (R59), fecal incontinence (R15), fever (R50), gait symptoms (ICD-10: R26), generalized hyperhidrosis (R61), headache (R51), hemorrhage (R58), illness (R69), lack of expected normal |

| **Predictors** | **Specific Variables Included** |
| --- | --- |
|  | physiological development (R62), malaise/fatigue (R53), other general symptoms/signs (R68), pain (R52), shock (R57), skin symptoms (ICD-10: R20-R23), symptoms and signs concerning food and fluid intake (R63), symptoms and signs specifically associated with systemic inflammation and infection (R65), syncope/collapse (R55), suicidal ideation (R45.851**)**, urinary incontinence (R39.81), urinary tract infection (N39.0) |
| Injury history | History of falls (ICD-10: Z91.81), history of fracture (ICD-10: Z87.81) |
| **Social and Medical Factors** |  |
| Health Screening and Medical Procedures | Depression screening (Z13), digital rectal exam (CPT G0102), X-ray (CPT 73090, 73060, 73030, 73100, 73110), head/neck computed tomography  (CPT 70450, 70470, 70480, 70486, 70487, 70490, 70491, and 70492),  Human Immunodeficiency Virus screening (CPT 86701, 86702, 86703,  87389, and G0435), medical nutrition therapy (CPT 97802, 97803, 97804, G0270, and G0271), pelvic/breast exam (CPT G0101), sexually transmitted infections testing (CPT 87491, 87591, 87661, 87801, 86631, 87110, 87270,  87320), swallowing evaluation and treatment (CPT 92610, 92526) |
| Social Determinants of  Health | Access to care issues (Z75), family conflict (Z62), marital problem (Z63), social environmental problems (Z60), housing and income problems (Z59), illiteracy (Z55), interpersonal relationship problems (Z63.0), primary support challenges (Z63.9) |
| Trauma History | History of physical abuse and rape (Z91) history of traumatic brain injury (Z87), counseling for victims of spousal abuse (Z69), history of emotional abuse (Z62), history of neglect (Z91.412), history of other abuse (Z91.49) |
| Health Screening and Medical Procedures | Depression screening (Z13), digital rectal exam (CPT G0102), X-ray (CPT 73090, 73060, 73030, 73100, 73110), head/neck computed tomography  (CPT 70450, 70470, 70480, 70486, 70487, 70490, 70491, and 70492),  Human Immunodeficiency Virus screening (CPT 86701, 86702, 86703,  87389, and G0435), medical nutrition therapy (CPT 97802, 97803, 97804, G0270, and G0271), pelvic/breast exam (CPT G0101), sexually transmitted infections testing (CPT 87491, 87591, 87661, 87801, 86631, 87110, 87270,  87320), swallowing evaluation and treatment (CPT 92610, 92526) |

**Note.** CPT=Current Procedural Terminology, ICD-10= International Classification of Diseases, Tenth Revision.

**Supplementary Table S3:** Baseline Chronic Conditions and Other Social and Medical Factors of the Total Cohort

| Baseline Conditions and Social/Medical Factors | Total |  |
| --- | --- | --- |
|  | N | % |
| **Chronic Conditions** | 2,261,166 | 100% |
| Acute Myocardial Infarction | 14,458 | 0.6% |
| Alzheimer's Disease^b^ | 71,084 | 3.1% |
| Alzheimer's Disease and Related Disorders or Senile Dementia | 187,805 | 8.3% |
| Atrial Fibrillation | 210,074 | 9.3% |
| Cataract | 550,509 | 24.3% |
| Chronic Kidney Disease | 483,204 | 21.4% |
| Chronic Obstructive Pulmonary Disease | 231,920 | 10.3% |
| Heart Failure | 275,375 | 12.2% |
| Diabetes | 643,463 | 28.5% |
| Glaucoma | 221,235 | 9.8% |
| Hip/Pelvic Fracture | 12,946 | 0.6% |
| Ischemic Heart Disease | 663,312 | 29.3% |
| Depression^b^ | 326,386 | 14.4% |
| Osteoporosis | 179,704 | 7.9% |
| Rheumatoid Arthritis/Osteoarthritis | 845,574 | 37.4% |
| Stroke / Transient Ischemic Attack | 78,506 | 3.5% |
| Breast Cancer | 91,129 | 4.0% |
| Colorectal Cancer | 27,178 | 1.2% |
| Prostate Cancer | 83,625 | 3.7% |
| Lung Cancer | 16,048 | 0.7% |
| Endometrial Cancer | 8,557 | 0.4% |
| Anemia | 482,929 | 21.4% |
| Asthma | 112,490 | 5.0% |
| Hyperlipidemia | 1,224,194 | 54.1% |
| Benign Prostatic Hyperplasia | 182,803 | 8.1% |
| Hypertension | 1,460,984 | 64.6% |
| Acquired Hypothyroidism | 205,463 | 9.1% |
| Attention-Deficit/Hyperactivity Disorder and Other Conduct Disorders | 8,537 | 0.4% |
| Alcohol Use Disorders | 28,718 | 1.3% |
| Anxiety Disorders | 307,163 | 13.6% |
| Autism^a^ | 596 | 0.0% |
| Bipolar Disorder | 34,418 | 1.5% |
| Traumatic Brain Injury and Nonpsychotic Mental Disorders due to Brain  Damage | 6,948 | 0.3% |
| Cerebral Palsy | 2,264 | 0.1% |

| Baseline Conditions and Social/Medical Factors | Total |  |
| --- | --- | --- |
|  | N | % |
| Cystic Fibrosis and Other Metabolic Developmental Disorders | 16,977 | 0.8% |
| Major Depressive Affective Disorder | 309,598 | 13.7% |
| Drug Use Disorder -Medicare Only Claims | 28,582 | 1.3% |
| Epilepsy | 34,233 | 1.5% |
| Fibromyalgia, Chronic Pain and Fatigue | 389,064 | 17.2% |
| Sensory - Deafness and Hearing Impairment | 138,400 | 6.1% |
| Viral Hepatitis (General) | 14,190 | 0.6% |
| Human Immunodeficiency Virus and/or Acquired Immunodeficiency  Syndrome^c^ | 2,416 | 0.1% |
| Intellectual Disabilities and Related Conditions | 7,105 | 0.3% |
| Learning Disabilities | 1,993 | 0.1% |
| Leukemia and Lymphomas | 37,667 | 1.7% |
| Liver Disease, Cirrhosis and Other Liver Conditions (excluding Hepatitis) | 76,593 | 3.4% |
| Migraine and other Chronic Headache | 53,026 | 2.3% |
| Mobility Impairments | 41,483 | 1.8% |
| Multiple Sclerosis and Transverse Myelitis | 7,453 | 0.3% |
| Muscular Dystrophy | 710 | 0.0% |
| Obesity | 328,085 | 14.5% |
| Other Developmental Delays^a^ | 855 | 0.0% |
| Overarching Opioid Use Disorder (Any of the Three Sub-Indicators) | 22,779 | 1.0% |
| Diagnosis and Procedure Basis for Opioid Use Disorder^b^ | 15,325 | 0.7% |
| Opioid-Related Hospitalization or Emergency Department^b^ | 11,969 | 0.5% |
| Use of Medication-Assisted Treatment^b^ | 1,623 | 0.1% |
| Personality Disorders | 19,815 | 0.9% |
| Post-Traumatic Stress Disorder | 7,406 | 0.3% |
| Peripheral Vascular Disease | 276,078 | 12.2% |
| Schizophrenia^b^ | 13,987 | 0.6% |
| Schizophrenia and Other Psychotic Disorders | 36,062 | 1.6% |
| Spina Bifida and Other Congenital Anomalies of the Nervous System^a^ | 1,621 | 0.1% |
| Spinal Cord Injury | 10,632 | 0.5% |
| Tobacco Use Disorders | 109,927 | 4.9% |
| Pressure Ulcers and Chronic Ulcers | 70,653 | 3.1% |
| Sensory - Blindness and Visual Impairment | 11,134 | 0.5% |
| Cerebral Palsy | 2,264 | 0.1% |
| Cystic Fibrosis and Other Metabolic Developmental Disorders | 16,977 | 0.8% |
| Major Depressive Affective Disorder | 309,598 | 13.7% |
| Drug Use Disorder -Medicare Only Claims | 28,582 | 1.3% |
| Epilepsy | 34,233 | 1.5% |
| Fibromyalgia, Chronic Pain and Fatigue | 389,064 | 17.2% |

| Baseline Conditions and Social/Medical Factors | Total |  |
| --- | --- | --- |
|  | N | % |
| Sensory - Deafness and Hearing Impairment | 138,400 | 6.1% |
| Viral Hepatitis (General) | 14,190 | 0.6% |
| Human Immunodeficiency Virus and/or Acquired Immunodeficiency Syndrome^c^ | 2,416 | 0.1% |
| Intellectual Disabilities and Related Conditions | 7,105 | 0.3% |
| Learning Disabilities | 1,993 | 0.1% |
| Leukemia and Lymphomas | 37,667 | 1.7% |
| Liver Disease, Cirrhosis and Other Liver Conditions (excluding Hepatitis) | 76,593 | 3.4% |
| Migraine and other Chronic Headache | 53,026 | 2.3% |
| Mobility Impairments | 41,483 | 1.8% |
| Multiple Sclerosis and Transverse Myelitis | 7,453 | 0.3% |
| Muscular Dystrophy | 710 | 0.0% |
| Obesity | 328,085 | 14.5% |
| Other Developmental Delays^a^ | 855 | 0.0% |
| Overarching Opioid Use Disorder (Any of the Three Sub-Indicators) | 22,779 | 1.0% |
| Diagnosis and Procedure Basis for Opioid Use Disorder^b^ | 15,325 | 0.7% |
| Opioid-Related Hospitalization or Emergency Department^b^ | 11,969 | 0.5% |
| Use of Medication-Assisted Treatment^b^ | 1,623 | 0.1% |
| Personality Disorders | 19,815 | 0.9% |
| Post-Traumatic Stress Disorder | 7,406 | 0.3% |
| Peripheral Vascular Disease | 276,078 | 12.2% |
| Schizophrenia^b^ | 13,987 | 0.6% |
| Schizophrenia and Other Psychotic Disorders | 36,062 | 1.6% |
| Spina Bifida and Other Congenital Anomalies of the Nervous System^a^ | 1,621 | 0.1% |
| Spinal Cord Injury | 10,632 | 0.5% |
| Tobacco Use Disorders | 109,927 | 4.9% |
| Pressure Ulcers and Chronic Ulcers | 70,653 | 3.1% |
| Sensory - Blindness and Visual Impairment | 11,134 | 0.5% |
| **Other Social and Medical Factors** |  |  |
| Head/Neck Computed Tomography Scan | 219,618 | 9.7% |
| X-ray | 180,947 | 8.0% |
| Pressure Ulcer^b^ | <11 | 0.0% |
| Pressure Ulcer Debridement^b^ | 23,090 | 1.0% |
| Depression Screening | 91,630 | 4.1% |
| Urinary Tract Infection Test^b^ | 280,205 | 12.4% |
| Pelvic/Breast Exam | 106,550 | 4.7% |
| Digital Rectal Exam^a^ | 6,906 | 0.3% |
| Medical Nutrition Therapy^a^ | 568 | 0.0% |
| Swallowing Evaluation and Treatment | 16,813 | 0.7% |

| Baseline Conditions and Social/Medical Factors | Total |  |
| --- | --- | --- |
|  | N | % |
| Sexually Transmitted Infections Testing | 10,119 | 0.4% |
| History of Traumatic Brain Injury | 2,692 | 0.1% |
| Demoralization and Apathy^a^ | 176 | 0.0% |
| Suicidal Ideation | 3,125 | 0.1% |
| Urinary Tract Infection Diagnosis | 347,282 | 15.4% |
| Urinary Incontinence | 2,331 | 0.1% |
| Fecal Incontinence | 12,626 | 0.6% |
| Abnormal Weight Loss | 70,763 | 3.1% |
| HIV Screening | 1,919 | 0.1% |
| History of Physical Abuse and Rape^a^ | 120 | 0.0% |
| History of Emotional Abuse^a^ | 48 | 0.0% |
| History of Neglect^a^ | 12 | 0.0% |
| History of other Abuse^a^ | 36 | 0.0% |
| Marital Problem | 62,591 | 2.8% |
| Counseling for the victim of spousal abuse^a^ | 14 | 0.0% |
| Family Conflict | 685 | 0.0% |
| Interpersonal Relationship Problems^a^ | <11 | 0.0% |
| Illiteracy | 312 | 0.0% |
| Housing and Income Problems | 1,826 | 0.1% |
| Social Environmental Problems | 3,197 | 0.1% |
| Primary Support Challenges | 6,768 | 0.3% |
| Access to Care Issues^a^ | 881 | 0.0% |

**Note.** ^a^not included in the analyses due to small sample sizes; ^b^variable combined with another variable: Alzheimer’s Disease -> under Alzheimer’s Disease and Related Dementias, Depression

-> under Depressive Disorders, Diagnosis and Procedure Basis for Opioid Use Disorder -> under Opioid Use Disorder, Opioid-Related Hospitalization or Emergency Department -> under Opioid Use Disorder, Use of Medication-Assisted Treatment -> under Opioid Use Disorder, Schizophrenia -> Schizophrenia and other Psychotic Disorders, Pressure Ulcer, Pressure Ulcer Debridement -> Pressure Ulcers (in Chronic Conditions), Urinary Tract Infections Testing -> Urinary Tract Infections Diagnosis; ^c^excluded due to collinearity. CPT= Current Procedural Terminology.

**Supplementary Table S4:** Baseline Characteristics of the Training, Validation, and Testing Cohorts

|  | Training (50%) | | Validation  (25%) | | Testing (25%) | |
| --- | --- | --- | --- | --- | --- | --- |
|  | N | % | N | % | N | % |
| Total | 1,130,302 | 100% | 565,688 | 100% | 565,176 | 100% |
| Elder Mistreatment Diagnosis |  |  |  |  |  |  |
| No | 1,128,041 | 99.8% | 564,472 | 99.8% | 564,005 | 99.8% |
| Yes | 2,261 | 0.2% | 1,216 | 0.2% | 1,171 | 0.2% |
| Sex |  |  |  |  |  |  |
| Male | 441,584 | 39.1% | 221,315 | 39.1% | 220,191 | 39.0% |
| Female | 688,718 | 60.9% | 344,373 | 60.9% | 344,985 | 61.0% |
| Race |  |  |  |  |  |  |
| Unknown | 14,031 | 1.2% | 7,011 | 1.2% | 7,084 | 1.3% |
| White | 958,607 | 84.8% | 479,316 | 84.7% | 478,738 | 84.7% |
| Black | 64,408 | 5.7% | 32,312 | 5.7% | 32,358 | 5.7% |
| Other | 8,204 | 0.7% | 4,088 | 0.7% | 4,108 | 0.7% |
| Asian/Pacific Islander | 31,181 | 2.8% | 15,682 | 2.8% | 15,803 | 2.8% |
| Hispanic | 49,980 | 4.4% | 25,262 | 4.5% | 25,157 | 4.5% |
| American Indian/Alaska Native | 3,891 | 0.3% | 2,017 | 0.4% | 1,928 | 0.3% |
| Medicaid Dual Eligible |  |  |  |  |  |  |
| No | 965,752 | 85.4% | 483,058 | 85.4% | 482,482 | 85.4% |
| Yes | 164,550 | 14.6% | 82,630 | 14.6% | 82,694 | 14.6% |
| Original Entitlement |  |  |  |  |  |  |
| Disability | 100,907 | 8.9% | 50,763 | 9.0% | 50,338 | 8.9% |
| Age 65+ | 1,029,395 | 91.1% | 514,925 | 91.0% | 514,838 | 91.1% |
| Age Group |  |  |  |  |  |  |
| 66-70 | 316,854 | 28.0% | 158,083 | 27.9% | 158,080 | 28.0% |
| 71-75 | 317,967 | 28.1% | 159,246 | 28.2% | 159,438 | 28.2% |
| 76-80 | 226,315 | 20.0% | 113,319 | 20.0% | 113,238 | 20.0% |
| 81-85 | 144,851 | 12.8% | 72,612 | 12.8% | 72,322 | 12.8% |
| 86+ | 124,315 | 11.0% | 62,428 | 11.0% | 62,098 | 11.0% |
| General Symptoms | 464,384 | 41.1% | 232,245 | 41.1% | 232,485 | 41.1% |
| Skin Symptoms | 141,164 | 12.5% | 71,054 | 12.6% | 70,755 | 12.5% |
| Gait Abnormalities | 186,591 | 16.5% | 93,101 | 16.5% | 92,997 | 16.5% |
| History of Fracture | 26,721 | 2.4% | 13,496 | 2.4% | 13,427 | 2.4% |
| History of Fall | 67,907 | 6.0% | 33,966 | 6.0% | 34,103 | 6.0% |
| Frailty |  |  |  |  |  |  |
| Score | 0.16 | 0.05 | 0.16 | 0.05 | 0.16 | 0.05 |
| Non-Frail (≤0.1) | 100,030 | 8.8% | 50,041 | 8.8% | 50,281 | 8.9% |

|  | Training (50%) | | Validation (25%) | | Testing (25%) | |
| --- | --- | --- | --- | --- | --- | --- |
|  | N | % | N | % | N | % |
| Pre-Frail (0.1-0.2) | 820,727 | 72.6% | 410,656 | 72.6% | 410,128 | 72.6% |
| Mild Frailty (0.2-0.3) | 181,461 | 16.1% | 91,035 | 16.1% | 90,653 | 16.0% |
| Moderate Frailty (0.3-0.4) | 26,806 | 2.4% | 13,338 | 2.4% | 13,496 | 2.4% |
| Severe Frailty (≥0.4) | 1,278 | 0.1% | 618 | 0.1% | 618 | 0.1% |
| Chronic Conditions |  |  |  |  |  |  |
| Acute Myocardial Infarction | 7,176 | 0.6% | 3,601 | 0.6% | 3,681 | 0.7% |
| Alzheimer's Disease^b^ | 35,492 | 3.1% | 17,733 | 3.1% | 17,859 | 3.2% |
| Alzheimer's Disease and Related  Disorders or Senile Dementia | 93,551 | 8.3% | 47,093 | 8.3% | 47,161 | 8.3% |
| Atrial Fibrillation | 105,172 | 9.3% | 52,608 | 9.3% | 52,294 | 9.3% |
| Cataract | 275,216 | 24.3% | 137,810 | 24.4% | 137,483 | 24.3% |
| Chronic Kidney Disease | 241,639 | 21.4% | 120,347 | 21.3% | 121,218 | 21.4% |
| Chronic Obstructive Pulmonary Disease | 115,742 | 10.2% | 58,203 | 10.3% | 57,975 | 10.3% |
| Heart Failure | 137,444 | 12.2% | 68,939 | 12.2% | 68,992 | 12.2% |
| Diabetes | 321,432 | 28.4% | 160,913 | 28.4% | 161,118 | 28.5% |
| Glaucoma | 110,761 | 9.8% | 55,308 | 9.8% | 55,166 | 9.8% |
| Hip/Pelvic Fracture | 6,389 | 0.6% | 3,296 | 0.6% | 3,261 | 0.6% |
| Ischemic Heart Disease | 331,246 | 29.3% | 166,417 | 29.4% | 165,649 | 29.3% |
| Depression^b^ | 162,873 | 14.4% | 81,761 | 14.5% | 81,752 | 14.5% |
| Osteoporosis | 89,675 | 7.9% | 45,007 | 8.0% | 45,022 | 8.0% |
| Rheumatoid Arthritis/Osteoarthritis | 422,693 | 37.4% | 211,179 | 37.3% | 211,702 | 37.5% |
| Stroke / Transient Ischemic Attack | 39,401 | 3.5% | 19,403 | 3.4% | 19,702 | 3.5% |
| Breast Cancer | 45,433 | 4.0% | 22,815 | 4.0% | 22,881 | 4.0% |
| Colorectal Cancer | 13,678 | 1.2% | 6,836 | 1.2% | 6,664 | 1.2% |
| Prostate Cancer | 41,722 | 3.7% | 21,160 | 3.7% | 20,743 | 3.7% |
| Lung Cancer | 8,006 | 0.7% | 4,001 | 0.7% | 4,041 | 0.7% |
| Endometrial Cancer | 4,309 | 0.4% | 2,125 | 0.4% | 2,123 | 0.4% |
| Anemia | 241,148 | 21.3% | 121,049 | 21.4% | 120,732 | 21.4% |
| Asthma | 56,279 | 5.0% | 28,057 | 5.0% | 28,154 | 5.0% |
| Hyperlipidemia | 611,779 | 54.1% | 306,252 | 54.1% | 306,163 | 54.2% |
| Benign Prostatic Hyperplasia | 91,539 | 8.1% | 45,809 | 8.1% | 45,455 | 8.0% |
| Hypertension | 730,363 | 64.6% | 365,272 | 64.6% | 365,349 | 64.6% |
| Acquired Hypothyroidism | 2,462 | 0.2% | 101,514 | 17.9% | 101,487 | 18.0% |
| Attention-Deficit/Hyperactivity Disorder and Other Conduct Disorders | 4,255 | 0.4% | 2,125 | 0.4% | 2,157 | 0.4% |
| Alcohol Use Disorders | 14,313 | 1.3% | 7,210 | 1.3% | 7,195 | 1.3% |
| Anxiety Disorders | 152,976 | 13.5% | 77,246 | 13.7% | 76,941 | 13.6% |
| Autism^b^ | 288 | 0.0% | 160 | 0.0% | 148 | 0.0% |

|  | Training (50%) | | Validation (25%) | | Testing (25%) | |
| --- | --- | --- | --- | --- | --- | --- |
|  | N | % | N | % | N | % |
| Bipolar Disorder | 17,119 | 1.5% | 8,630 | 1.5% | 8,669 | 1.5% |
| Traumatic Brain Injury and Nonpsychotic Mental Disorders due to Brain Damage | 3,426 | 0.3% | 1,713 | 0.3% | 1,809 | 0.3% |
| Cerebral Palsy | 1,126 | 0.1% | 566 | 0.1% | 572 | 0.1% |
| Cystic Fibrosis and Other Metabolic  Developmental Disorders | 8,490 | 0.8% | 4,242 | 0.7% | 4,245 | 0.8% |
| Major Depressive Affective Disorder | 154,387 | 13.7% | 77,779 | 13.7% | 77,432 | 13.7% |
| Drug Use Disorder -Medicare Only  Claims | 14,356 | 1.3% | 7,031 | 1.2% | 7,195 | 1.3% |
| Epilepsy | 17,144 | 1.5% | 8,646 | 1.5% | 8,443 | 1.5% |
| Fibromyalgia, Chronic Pain and Fatigue | 194,585 | 17.2% | 97,017 | 17.2% | 97,462 | 17.2% |
| Sensory - Deafness and Hearing  Impairment | 69,070 | 6.1% | 34,711 | 6.1% | 34,619 | 6.1% |
| Viral Hepatitis (General) | 7,055 | 0.6% | 3,586 | 0.6% | 3,549 | 0.6% |
| Human Immunodeficiency Virus/Acquired Immunodeficiency  Syndrome^c^ | 1,217 | 0.1% | 586 | 0.1% | 613 | 0.1% |
| Intellectual Disabilities and Related Conditions | 3,480 | 0.3% | 1,785 | 0.3% | 1,840 | 0.3% |
| Learning Disabilities | 1,020 | 0.1% | 506 | 0.1% | 467 | 0.1% |
| Leukemia and Lymphomas | 18,736 | 1.7% | 9,421 | 1.7% | 9,510 | 1.7% |
| Liver Disease, Cirrhosis and Other Liver Conditions (excluding Hepatitis) | 38,211 | 3.4% | 19,171 | 3.4% | 19,211 | 3.4% |
| Migraine and other Chronic Headache | 26,492 | 2.3% | 13,250 | 2.3% | 13,284 | 2.4% |
| Mobility Impairments | 20,770 | 1.8% | 10,341 | 1.8% | 10,372 | 1.8% |
| Multiple Sclerosis and Transverse  Myelitis | 3,829 | 0.3% | 1,802 | 0.3% | 1,822 | 0.3% |
| Muscular Dystrophy | 352 | 0.0% | 190 | 0.0% | 168 | 0.0% |
| Obesity | 164,089 | 14.5% | 81,855 | 14.5% | 82,141 | 14.5% |
| Other Developmental Delays^a^ | 417 | 0.0% | 215 | 0.0% | 223 | 0.0% |
| Overarching Opioid Use Disorder (Any  of the Three Sub-Indicators) | 11,395 | 1.0% | 5,641 | 1.0% | 5,743 | 1.0% |
| Diagnosis and Procedure Basis for Opioid Use Disorder^b^ | 7,661 | 0.7% | 3,785 | 0.7% | 3,879 | 0.7% |
| Opioid-Related Hospitalization or  Emergency Department^b^ | 6,040 | 0.5% | 2,962 | 0.5% | 2,967 | 0.5% |
| Use of Medication-Assisted Treatment^b^ | 813 | 0.1% | 377 | 0.1% | 433 | 0.1% |
| Personality Disorders | 9,827 | 0.9% | 4,928 | 0.9% | 5,060 | 0.9% |

|  | Training (50%) | | Validation (25%) | | Testing (25%) | |
| --- | --- | --- | --- | --- | --- | --- |
|  | N | % | N | % | N | % |
| Post-Traumatic Stress Disorder | 3,582 | 0.3% | 1,946 | 0.3% | 1,878 | 0.3% |
| Peripheral Vascular Disease | 137,971 | 12.2% | 69,368 | 12.3% | 68,739 | 12.2% |
| Schizophrenia^b^ | 7,032 | 0.6% | 3,525 | 0.6% | 3,430 | 0.6% |
| Schizophrenia and Other Psychotic  Disorders | 18,053 | 1.6% | 9,048 | 1.6% | 8,961 | 1.6% |
| Spina Bifida and Other Congenital Anomalies of the Nervous System^a^ | 782 | 0.1% | 427 | 0.1% | 412 | 0.1% |
| Spinal Cord Injury | 5,296 | 0.5% | 2,685 | 0.5% | 2,651 | 0.5% |
| Tobacco Use Disorders | 54,794 | 4.8% | 27,581 | 4.9% | 27,552 | 4.9% |
| Pressure Ulcers and Chronic Ulcers | 35,205 | 3.1% | 17,610 | 3.1% | 17,838 | 3.2% |
| Sensory - Blindness and Visual  Impairment | 5,635 | 0.5% | 2,752 | 0.5% | 2,747 | 0.5% |
| **Other Social and Medical Factors** |  |  |  |  |  |  |
| Head/Neck Computed Tomography Scan | 109,538 | 9.7% | 55,107 | 9.7% | 54,973 | 9.7% |
| X-ray | 90,610 | 8.0% | 44,981 | 8.0% | 45,356 | 8.0% |
| Pressure Ulcer^b^ | <11 | 0.0% | <11 | 0.0% | <11 | 0.0% |
| Pressure Ulcer Debridement^b^ | 11,499 | 1.0% | 5,726 | 1.0% | 5,865 | 1.0% |
| Depression Screening | 45,727 | 4.0% | 22,984 | 4.1% | 22,919 | 4.1% |
| Urinary Tract Infection Testing^b^ | 140,342 | 12.4% | 69,763 | 12.3% | 70,100 | 12.4% |
| Pelvic/Breast Exam | 53,413 | 4.7% | 26,291 | 4.6% | 26,846 | 4.8% |
| Digital Rectal Exam^a^ | 3,519 | 0.3% | 1,717 | 0.3% | 1,670 | 0.3% |
| Medical Nutrition Therapy^a^ | 277 | 0.0% | 162 | 0.0% | 129 | 0.0% |
| Swallowing Evaluation and Treatment | 8,425 | 0.7% | 4,214 | 0.7% | 4,174 | 0.7% |
| Sexually Transmitted Infections Testing | 5,050 | 0.4% | 2,519 | 0.4% | 2,550 | 0.5% |
| History of Traumatic Brain Injury | 1,306 | 0.1% | 700 | 0.1% | 686 | 0.1% |
| Demoralization and Apathy^a^ | 86 | 0.0% | 51 | 0.0% | 39 | 0.0% |
| Suicidal Ideation | 1,582 | 0.1% | 771 | 0.1% | 772 | 0.1% |
| Urinary Tract Infection Diagnosis | 173,756 | 15.4% | 86,854 | 15.4% | 86,672 | 15.3% |
| Urinary Incontinence | 1,162 | 0.1% | 568 | 0.1% | 601 | 0.1% |
| Fecal Incontinence | 6,312 | 0.6% | 3,174 | 0.6% | 3,140 | 0.6% |
| Abnormal Weight Loss | 35,317 | 3.1% | 17,657 | 3.1% | 17,789 | 3.1% |
| Human Immunodeficiency Virus  Screening | 966 | 0.1% | 477 | 0.1% | 476 | 0.1% |
| History of Physical Abuse and Rape^a^ | 75 | 0.0% | 27 | 0.0% | 18 | 0.0% |
| History of Emotional Abuse^a^ | 27 | 0.0% | <11 | 0.0% | <11 | 0.0% |
| History of Neglect^a^ | <11 | 0.0% | <11 | 0.0% | <11 | 0.0% |
| History of other Abuse^a^ | 11 | 0.0% | 12 | 0.0% | 13 | 0.0% |
| Marital Problem | 31,154 | 2.8% | 15,746 | 2.8% | 15,691 | 2.8% |

|  | Training (50%) | | Validation (25%) | | Testing (25%) | |
| --- | --- | --- | --- | --- | --- | --- |
|  | N | % | N | % | N | % |
| Counseling for the victim of spousal  abuse^a^ | <11 | 0.0% | <11 | 0.0% | <11 | 0.0% |
| Family Conflict | 366 | 0.0% | 150 | 0.0% | 169 | 0.0% |
| Interpersonal Relationship Problems^a^ | <11 | 0.0% | <11 | 0.0% | <11 | 0.0% |
| Illiteracy | 168 | 0.0% | 78 | 0.0% | 66 | 0.0% |
| Housing and Income Problems | 928 | 0.1% | 410 | 0.1% | 488 | 0.1% |
| Social Environmental Problems | 1,624 | 0.1% | 778 | 0.1% | 795 | 0.1% |
| Primary Support Challenges | 3,392 | 0.3% | 1,645 | 0.3% | 1,731 | 0.3% |
| Access to Care Issues^a^ | 446 | 0.0% | 236 | 0.0% | 199 | 0.0% |

**Note.** ^a^not included in the analyses due to small sample sizes; ^b^variable combined with another variable: Alzheimer’s Disease -> under Alzheimer’s Disease and Related Dementias, Depression

-> under Depressive Disorders, Diagnosis and Procedure Basis for Opioid Use Disorder -> under Opioid Use Disorder, Opioid-Related Hospitalization or Emergency Department -> under Opioid Use Disorder, Use of Medication-Assisted Treatment -> under Opioid Use Disorder, Schizophrenia -> Schizophrenia and other Psychotic Disorders, Pressure Ulcer, Pressure Ulcer Debridement -> Pressure Ulcers (in Chronic Conditions), Urinary Tract Infections Testing -> Urinary Tract Infections Diagnosis; ^c^excluded due to collinearity.

**Supplementary Table S5:** Logistic Regression Results for the Full Prediction Model of Elder Mistreatment Within 2 years

|  | **Variable** | **Level** | **Reference** | **OR (95% CI)** |
| --- | --- | --- | --- | --- |
| **Demographics** | Sex | Male | Female | 0.98 (0.86, 1.12) |
|  | Race | Unknown |  | 0.93 (0.54, 1.62) |
|  |  | Black |  | **1.32 (1.09, 1.58)** |
|  |  | Other | White | 1.12 (0.63, 1.98) |
|  |  | Asian |  | 0.94 (0.67, 1.31) |
|  |  | Hispanic |  | 1.09 (0.87, 1.36) |
|  |  | AI/AN |  | 0.90 (0.42, 1.89) |
|  | Medicaid Dual Eligibility | Yes | No | **1.54 (1.35, 1.75)** |
|  | Original Medicare Entitlement | Disability | Age 65+ | **1.39 (1.20, 1.60)** |
|  | Age | 71-75 |  | 1.00 (0.87, 1.15) |
|  |  | 76-80 | ≤70 | 1.07 (0.92, 1.25) |
|  |  | 81-85 |  | 1.05 (0.88, 1.26) |
|  |  | 85+ |  | 1.04 (0.86, 1.26) |
| **CCW**  **Chronic Conditions** | Acute Myocardial Infarction | Yes | No | 0.99 (0.61, 1.59) |
|  | Alzheimer’s Disease and  Related Dementias | Yes | No | 0.95 (0.80, 1.13) |
|  | Atrial Fibrillation | Yes | No | 0.94 (0.80, 1.11) |
|  | Cataracts | Yes | No | **0.80 (0.70, 0.91)** |
|  | Chronic Kidney Disease | Yes | No | **1.29 (1.14, 1.47)** |
|  | Chronic Obstructive Pulmonary  Disease | Yes | No | 1.15 (1.00, 1.33) |
|  | Congestive Heart Failure | Yes | No | 1.05 (0.91, 1.22) |
|  | Diabetes | Yes | No | 0.95 (0.84, 1.07) |
|  | Glaucoma | Yes | No | 1.04 (0.88, 1.24) |
|  | Hip Fracture | Yes | No | **0.43 (0.22, 0.82)** |
|  | Ischemic Heart Disease | Yes | No | 0.92 (0.81, 1.04) |
|  | Osteoporosis | Yes | No | 0.87 (0.73, 1.04) |
|  | Rheumatoid Arthritis /  Osteoarthritis | Yes | No | 0.92 (0.82, 1.03) |
|  | Stroke/Transient Ischemic  Attack | Yes | No | 0.92 (0.72, 1.16) |
|  | Cancer Breast | Yes | No | 1.13 (0.90, 1.42) |
|  | Cancer Colorectal | Yes | No | 0.84 (0.54, 1.30) |
|  | Cancer Prostate | Yes | No | 0.86 (0.63, 1.17) |
|  | Cancer Lung | Yes | No | **1.93 (1.34, 2.78)** |
|  | Cancer Endometrial | Yes | No | 1.14 (0.59, 2.20) |
|  | Anemia | Yes | No | 0.92 (0.81, 1.04) |
|  | Asthma | Yes | No | 1.07 (0.88, 1.29) |
|  | Hyperlipidemia | Yes | No | **0.80 (0.72, 0.90)** |

|  | Benign Prostatic Hyperplasia | Yes | No | 0.90 (0.73, 1.11) |
| --- | --- | --- | --- | --- |
|  | Hypertension | Yes | No | **0.84 (0.74, 0.96)** |
|  | Hypothyroidism | Yes | No | 1.09 (0.96, 1.23) |
|  | Attention-Deficit/Hyperactivity  Disorder | Yes | No | 1.04 (0.63, 1.70) |
|  | Alcohol Use Disorders | Yes | No | **1.64 (1.26, 2.14)** |
|  | Anxiety | Yes | No | **1.36 (1.19, 1.55)** |
|  | Bipolar Disorders | Yes | No | **1.31 (1.03, 1.66)** |
|  | Brain Injury | Yes | No | 1.38 (0.83, 2.31) |
|  | Cerebral Palsy | Yes | No | 0.32 (0.05, 2.33) |
|  | Cystic Fibrosis | Yes | No | 1.05 (0.67, 1.64) |
|  | Depressive Disorders | Yes | No | **1.23 (1.07, 1.42)** |
|  | Drug Use Disorders | Yes | No | 1.04 (0.74, 1.46) |
|  | Epilepsy | Yes | No | 0.99 (0.74, 1.32) |
|  | Fibromyalgia | Yes | No | 1.06 (0.93, 1.21) |
|  | Hearing Impairment | Yes | No | 1.15 (0.96, 1.38) |
|  | Viral Hepatitis | Yes | No | **1.47 (1.01, 2.14)** |
|  | Intellectual Disabilities | Yes | No | 0.62 (0.30, 1.27) |
|  | Learning Disabilities | Yes | No | **2.35 (1.15, 4.78)** |
|  | Leukemia/Lymphoma | Yes | No | **1.47 (1.08, 2.01)** |
|  | Liver Disease | Yes | No | **1.51 (1.24, 1.84)** |
|  | Migraine | Yes | No | 0.82 (0.60, 1.10) |
|  | Mobility Impairment | Yes | No | 1.11 (0.85, 1.46) |
|  | Multiple Sclerosis | Yes | No | 0.53 (0.22, 1.29) |
|  | Muscular Dystrophy | Yes | No | 2.62 (0.64, 10.64) |
|  | Obesity | Yes | No | 1.07 (0.93, 1.22) |
|  | Opioid Use Disorder | Yes | No | 0.90 (0.60, 1.33) |
|  | Personality Disorders | Yes | No | **1.55 (1.16, 2.08)** |
|  | Post-Traumatic Stress Disorder | Yes | No | 1.11 (0.69, 1.78) |
|  | Peripheral Vascular Disease | Yes | No | 0.88 (0.77, 1.02) |
|  | Schizophrenia and other  Psychotic Disorders | Yes | No | 1.14 (0.89, 1.44) |
|  | Spinal Cord Injury | Yes | No | 0.90 (0.53, 1.53) |
|  | Tobacco Use | Yes | No | 1.20 (1.00, 1.45) |
|  | Ulcers | Yes | No | **1.36 (1.11, 1.66)** |
|  | Visual Impairment | Yes | No | 0.69 (0.39, 1.23) |
| **Symptoms** | General | Yes | No | **1.39 (1.23, 1.57)** |
|  | Skin | Yes | No | **1.19 (1.04, 1.35)** |
|  | Gait Abnormality | Yes | No | 1.13 (0.99, 1.29) |
|  | History of Fracture | Yes | No | 1.04 (0.79, 1.36) |
|  | History of Fall | Yes | No | 1.19 (0.99, 1.44) |
|  | Frailty Score | Per 0.01 Increase | | **1.04 (1.03, 1.06)** |

| **Other Social and Medical Factors** | Head/Neck Computed Tomography Scan | Yes | No | 1.02 (0.87, 1.19) |
| --- | --- | --- | --- | --- |
|  | X-ray | Yes | No | 0.94 (0.80, 1.12) |
|  | Depression Screening | Yes | No | **0.72 (0.53, 0.97)** |
|  | Pelvic/Breast Exam | Yes | No | 0.94 (0.71, 1.24) |
|  | Swallowing Evaluation &  Treatment | Yes | No | 0.77 (0.52, 1.13) |
|  | Sexually Transmitted Infections  Testing | Yes | No | **2.25 (1.44, 3.53)** |
|  | History of Traumatic Brain  Injury | Yes | No | 0.87 (0.35, 2.15) |
|  | Suicidal Ideation | Yes | No | 0.67 (0.35, 1.27) |
|  | Urinary Tract Infection | Yes | No | 0.88 (0.77, 1.00) |
|  | Urinary Incontinence | Yes | No | 1.00 (0.32, 3.11) |
|  | Fecal Incontinence | Yes | No | 1.50 (0.98, 2.30) |
|  | Abnormal Weight Loss | Yes | No | **1.26 (1.03, 1.55)** |
|  | Human Immunodeficiency  Virus Screening | Yes | No | 1.77 (0.65, 4.76) |
|  | Marital Problems | Yes | No | **1.59 (1.25, 2.01)** |
|  | Family Conflict | Yes | No | 0.94 (0.32, 2.80) |
|  | Illiteracy | Yes | No | 1.85 (0.24, 14.51) |
|  | Housing and Income Problems | Yes | No | **2.70 (1.59, 4.61)** |
|  | Social Environmental Problems | Yes | No | **1.91 (1.09, 3.35)** |
|  | Primary Support Challenges | Yes | No | **3.55 (2.36, 5.34)** |

**Note.** AI/AN=American Indian/Alaska Native; CCW=Chronic Conditions Warehouse; CI=confidence interval; EM=elder mistreatment; OR=odds ratio.

**Supplementary Table S6:** Predicted Versus Observed Probabilities of Elder Mistreatment (EM) by Deciles

| Decile | Predicted | Observed | EM Events |
| --- | --- | --- | --- |
| 1 | 0.000517 | 0.000531 | 30 |
| 2 | 0.000664 | 0.000619 | 35 |
| 3 | 0.000742 | 0.000372 | 21 |
| 4 | 0.000818 | 0.000672 | 38 |
| 5 | 0.00092 | 0.001026 | 58 |
| 6 | 0.001045 | 0.001044 | 59 |
| 7 | 0.001213 | 0.001132 | 64 |
| 8 | 0.001479 | 0.001575 | 89 |
| 9 | 0.001995 | 0.002406 | 136 |
| 10 | 0.004816 | 0.00483 | 273 |

**Supplementary Table S7:** Elder Mistreatment (EM) Logistic Model Specification

| Cutpoint | |  | EM - Observed | |  |  | | | |
| --- | --- | --- | --- | --- | --- | --- | --- | --- | --- |
| Percentile | Number | Yes | | No |  | sensitivity | specificity | PPV | NPV |
| Logistic Regression - Full Model | 0.0014^a^ | Yes | 488 | 155,369 |  | 0.608 | 0.725 | 0.003 | 0.999 |
|  |  | No | 315 | 409,004 |  |  |  |  |  |
| 1 | 0.0004225 | Yes | 801 | 558,723 |  | 0.998 | 0.010 | 0.001 | 1.000 |
|  |  | No | 2 | 5,650 |  |  |  |  |  |
| 5 | 0.0005542 | Yes | 789 | 536,128 |  | 0.983 | 0.050 | 0.001 | 1.000 |
|  |  | No | 14 | 28,245 |  |  |  |  |  |
| 25 | 0.0007424 | Yes | 728 | 423,153 |  | 0.907 | 0.250 | 0.002 | 0.999 |
|  |  | No | 75 | 141,220 |  |  |  |  |  |
| 30 | 0.0007788 | Yes | 717 | 394,907 |  | 0.893 | 0.300 | 0.002 | 0.999 |
|  |  | No | 86 | 169,466 |  |  |  |  |  |
| 40 | 0.0008651 | Yes | 679 | 338,427 |  | 0.846 | 0.400 | 0.002 | 0.999 |
|  |  | No | 124 | 225,946 |  |  |  |  |  |
| 50 | 0.0009776 | Yes | 621 | 281,967 |  | 0.773 | 0.500 | 0.002 | 0.999 |
|  |  | No | 182 | 282,406 |  |  |  |  |  |
| 60 | 0.0011194 | Yes | 562 | 225,502 |  | 0.700 | 0.600 | 0.002 | 0.999 |
|  |  | No | 241 | 338,871 |  |  |  |  |  |
| 70 | 0.0013214 | Yes | 498 | 169,040 |  | 0.620 | 0.700 | 0.003 | 0.999 |
|  |  | No | 305 | 395,333 |  |  |  |  |  |
| 75 | 0.0014714 | Yes | 464 | 140,840 |  | 0.578 | 0.750 | 0.003 | 0.999 |
|  |  | No | 339 | 423,533 |  |  |  |  |  |
| 95 | 0.0035710 | Yes | 186 | 28,073 |  | 0.232 | 0.950 | 0.007 | 0.999 |
|  |  | No | 617 | 536,300 |  |  |  |  |  |
| 99 | 0.0079391 | Yes | 65 | 5,587 |  | 0.081 | 0.990 | 0.012 | 0.999 |
|  |  | No | 738 | 558,786 |  |  |  |  |  |

**Note.** ^a^Cutpoint selected by Youden index, PPV=positive predictive value; NPV=negative predictive value.

**Supplementary Table S8:** Model C-statistic Results for Sex, Race, and Medicaid Dual Eligibility Interactions

C-statistic

| Sex | Race | Medicaid | N EM % | All Variables | Manual Selection | Stepwise Selection |
| --- | --- | --- | --- | --- | --- | --- |
|  |  |  | 1,130,30 | 0.72 |  | 0.72 |

Overall

2 1,551 0.14% (0.71, 0.74) N/A

(0.71, 0.73)

| Male | Other Racial Groups^a^ | No Medicaid | 43,329 | 34 | 0.08% | 0.85  (0.78, 0.92) | 0.82  (0.74, 0.90) | 0.76  (0.66, 0.86) |
| --- | --- | --- | --- | --- | --- | --- | --- | --- |
|  |  | Medicaid | 23,901 | 70 | 0.29% | 0.85  (0.80, 0.89) | 0.82  (0.76, 0.87) | 0.75  (0.69, 0.81) |
|  | NHW | No Medicaid | 346,758 | 325 | 0.09% | 0.75  (0.72, 0.78) | 0.73  (0.70, 0.76) | 0.73  (0.70, 0.76) |
|  |  | Medicaid | 27,596 | 100 | 0.36% | 0.76  (0.71, 0.82) | 0.74  (0.69, 0.80) | 0.68  (0.62, 0.74) |
| Femal | Other Racial Groups^a^ | No Medicaid | 57,180 | 83 | 0.15% | 0.82  (0.78, 0.87) | 0.79  (0.74, 0.84) | 0.70  (0.65, 0.76) |
|  |  | Medicaid | 47,285 | 118 | 0.25% | 0.78  (0.73, 0.82) | 0.74  (0.69, 0.79) | 0.78  (0.67, 0.77) |
| e |  | No Medicaid | 518,485 | 639 | 0.12% | 0.70  (0.68, 0.72) | 0.68  (0.66, 0.70) | 0.69  (0.66, 0.71) |
|  |  |  |  |  |  |  |  |  |

**Note.** ^a^Includes American Indian/Alaska Native, Asian, Black, Hispanic, Other, and Unknown, EM=elder mistreatment; NHW=Non-Hispanic White.

| NHW | 0.73 | 0.68 | 0.66 |
| --- | --- | --- | --- |
| Medicaid | 65,768 182 0.28% (0.69, 0.77) | (0.64, 0.72) | (0.62, 0.70) |

**Supplementary Table S9:** Factors Associated with Elder Mistreatment by Sex

|  | Male |  |  |
| --- | --- | --- | --- |
| Risk Factors - OR (95% CI) | | Protective Factors - OR (95% CI) | |
| Age 76-80 (vs 66-70) | 1.40 (1.08, 1.81) | Atrial Fibrillation | 0.72 (0.55,  0.95) |
| Age 81-85 (vs 66-70) | 1.45 (1.07, 1.96) | Cataracts | 0.69 (0.55,  0.86) |
| Alcohol Use Disorders | 1.82 (1.28, 2.58) | Hyperlipidemia | 0.71 (0.59,  0.87) |
| Anxiety | 1.35 (1.05, 1.73) | Peripheral Vascular  Disease | 0.75 (0.59,  0.96) |
| Chronic Kidney Disease | 1.48 (1.21, 1.83) | Suicidal Ideation | 0.19 (0.04,  0.84) |
| Chronic Obstructive Pulmonary  Disease | 1.46 (1.16, 1.84) |  |  |
| Disability Entitlement (vs Old  Age) | 1.64 (2.06, 1.31) |  |  |
| Frailty Score (0.01 increase) | 1.06 (1.03, 1.09) |  |  |
| General | 1.40 (1.14, 1.73) |  |  |
| Housing and Income Problems | 3.84 (1.86, 7.92) |  |  |
| Leukemia/Lymphoma | 1.90 (1.26, 2.88) |  |  |
| Marital Problem | 1.88 (1.29, 2.75) |  |  |
| Muscular Dystrophy | 6.67 (1.57,  28.30) |  |  |
| Primary Support Issues | 2.81 (1.02, 7.77) |  |  |
| Tobacco Use | 1.50 (1.15, 1.97) |  |  |
| Ulcers | 1.79 (1.32, 2.42) |  |  |
| Viral Hepatitis | 1.86 (1.11, 3.12) |  |  |
|  | Female |  |  |
| Risk Factors - OR (95% CI) | | Protective Factors - OR (95% CI) | |
| Abnormal Weight Loss | 1.45 (1.13, 1.85) | Anemia | 0.85 (0.73,  0.99) |
| Anxiety | 1.37 (1.17, 1.60) | Cataracts | 0.83 (0.71,  0.97) |
| Cancer Lung | 1.97 (1.24, 3.13) | Depression Screening | 0.66 (0.45,  0.96) |
| Chronic Kidney Disease | 1.19 (1.02, 1.40) | Hip Fracture | 0.26 (0.11,  0.65) |
| Depression | 1.27 (1.08, 1.50) | Hyperlipidemia | 0.82 (0.71,  0.94) |
| Disability Entitlement (vs Age  65+) | 1.48 (1.77, 1.24) |  |  |
| Falls | 1.29 (1.04, 1.60) |  |  |
| Fibromyalgia | 1.18 (1.01, 1.37) |  |  |
| Frailty Score (0.01 increase) | 1.04 (1.02, 1.06) |  |  |

| Gait Abnormality | 1.21 (1.03, 1.42) |
| --- | --- |
| General | 1.36 (1.18, 1.58) |
| Learning Disabilities | 2.49 (1.01, 6.14) |
| Liver Disease | 1.58 (1.23, 2.02) |
| Marital Problem | 1.42 (1.04, 1.92) |
| Personality Disorders | 1.57 (1.11, 2.20) |
| Primary Support Issues | 3.63 (2.32, 5.68) |
| Social Environmental Problems | 2.28 (1.23, 4.23) |
| Sexually Transmitted Infections  Testing | 2.63 (1.62, 4.29) |

**Supplementary Table S10:** Factors Associated with Elder Mistreatment by Race

| White | | | |
| --- | --- | --- | --- |
| Risk Factors - OR (95% CI) | | Protective Factors - OR (95% CI) | |
| Alcohol Use Disorders | 1.52 (1.12, 2.06) | Cataracts | 0.84 (0.73, 0.96) |
| Anxiety | 1.36 (1.17, 1.57) | Depression  Screening | 0.68 (0.49, 0.96) |
| Bipolar Disorders | 1.46 (1.12, 1.90) | Hip Fracture | 0.43 (0.21, 0.84) |
| Cancer Lung | 1.82 (1.22, 2.71) | Hyperlipidemia | 0.79 (0.70, 0.90) |
| Chronic Kidney Disease | 1.29 (1.12, 1.48) | Rheumatoid  Arthritis/ Osteoarthritis | 0.87 (0.77, 0.99) |
| Depression | 1.22 (1.04, 1.42) |  |  |
| Disability Entitlement  (vs Age 65+) | 1.39 (1.64, 1.17) |  |  |
| Frailty Score (0.01  increase) | 1.04 (1.03, 1.06) |  |  |
| General | 1.40 (1.22, 1.60) |  |  |
| HIV Screening | 3.30 (1.21, 8.96) |  |  |
| Housing and Income  Problems | 2.63 (1.35, 5.11) |  |  |
| Learning Disabilities | 3.08 (1.51, 6.30) |  |  |
| Liver Disease | 1.55 (1.24, 1.93) |  |  |
| Marital Problem | 1.60 (1.23, 2.08) |  |  |
| Personality Disorders | 1.46 (1.05, 2.03) |  |  |
| Primary Support Issues | 3.40 (2.15, 5.36) |  |  |
| Skin | 1.18 (1.02, 1.37) |  |  |
| Tobacco Use | 1.36 (1.10, 1.66) |  |  |
| Ulcers | 1.26 (1.01, 1.58) |  |  |
| Viral Hepatitis | 2.04 (1.31, 3.16) |  |  |
| Other Racial Groups | | | |
| Risk Factors - OR (95% CI) | | Protective Factors - OR (95% CI) | |
| Alcohol Use Disorders | 2.14 (1.26, 3.65) | Anemia | 0.65 (0.49, 0.86) |
| Anxiety | 1.43 (1.04, 1.95) | Benign Prostatic  Hyperplasia | 0.55 (0.33, 0.94) |
| Brain Injury | 3.42 (1.43, 8.15) | Cataracts | 0.58 (0.41, 0.81) |
| Disability Entitlement  (vs Old Age) | 1.93 (2.51, 1.48) | Hyperlipidemia | 0.76 (0.59, 0.98) |
| Drug Use Disorders | 2.25 (1.24, 4.10) | Hypertension | 0.73 (0.54, 0.98) |
| Frailty Score (0.01  increase) | 1.05 (1.02, 1.09) | X-ray | 0.51 (0.32, 0.83) |
| General | 1.37 (1.04, 1.81) |  |  |
| Housing and Income  Problems | 5.01 (2.03, 12.33) |  |  |
| Personality Disorders | 2.15 (1.13, 4.10) |  |  |

| Primary Support Issues | 4.26 (1.70, 10.70) |
| --- | --- |
| Sexually Transmitted  Infections Testing | 4.38 (2.40, 8.00) |
| Ulcers | 1.82 (1.20, 2.76) |

**Supplementary Table S11:** Variable Importance Metrics for the Random Forest and Gradient Boosting Artificial Intelligence/Machine Learning Methods

| **Variable** | **Random Forest** | **RF**  **Rank** | **Gradient Boosting** | **GB Rank** |
| --- | --- | --- | --- | --- |
| Frailty Score | 0.053 | 1 | 0.059 | 1 |
| Race | 0.029 | 3 | 0.038 | 2 |
| Chronic Kidney Disease | 0.020 | 4 | 0.032 | 3 |
| Hypertension | 0.014 | 21 | 0.026 | 4 |
| Abnormal Weight Loss | 0.016 | 9 | 0.023 | 5 |
| Gait Abnormality | 0.015 | 12 | 0.023 | 6 |
| Alzheimer’s Disease and Related  Dementias | 0.011 | 41 | 0.023 | 7 |
| Glaucoma | 0.007 | 62 | 0.023 | 8 |
| Anemia | 0.014 | 20 | 0.022 | 9 |
| Age | 0.039 | 2 | 0.021 | 10 |
| Atrial Fibrillation | 0.016 | 10 | 0.021 | 11 |
| Rheumatoid Arthritis/Osteoarthritis | 0.013 | 28 | 0.020 | 12 |
| Attention-Deficit/Hyperactivity Disorder | 0.011 | 38 | 0.019 | 13 |
| Medicaid Dual Eligibility | 0.009 | 57 | 0.019 | 14 |
| Cancer Colorectal | 0.008 | 58 | 0.019 | 15 |
| Cystic Fibrosis | 0.017 | 7 | 0.018 | 16 |
| Osteoporosis | 0.014 | 19 | 0.018 | 17 |
| Cataracts | 0.012 | 31 | 0.018 | 18 |
| Congestive Heart Failure | 0.011 | 44 | 0.018 | 19 |
| Viral Hepatitis | 0.016 | 11 | 0.017 | 20 |
| Cancer Prostate | 0.007 | 67 | 0.016 | 21 |
| Depressive Disorders | 0.015 | 16 | 0.015 | 22 |
| Epilepsy | 0.013 | 29 | 0.015 | 23 |
| Personality Disorders | 0.012 | 33 | 0.015 | 24 |
| Leukemia/Lymphoma | 0.003 | 80 | 0.014 | 25 |
| General | 0.011 | 46 | 0.013 | 26 |
| Benign Prostatic Hyperplasia | 0.010 | 49 | 0.013 | 27 |
| Ulcers | 0.016 | 8 | 0.012 | 28 |
| Alcohol Use Disorders | 0.012 | 30 | 0.012 | 29 |
| Ischemic Heart Disease | 0.011 | 40 | 0.011 | 30 |
| Post-Traumatic Stress Disorder | 0.005 | 74 | 0.011 | 31 |
| Sexually Transmitted Infections Testing | 0.004 | 77 | 0.011 | 32 |
| Acute Myocardial Infarction | 0.004 | 79 | 0.011 | 33 |
| Fibromyalgia | 0.015 | 13 | 0.010 | 34 |
| History of Fall | 0.015 | 15 | 0.010 | 35 |
| Stroke/ Transient Ischemic Attack | 0.013 | 27 | 0.010 | 36 |
| Schizophrenia | 0.012 | 35 | 0.010 | 37 |
| Housing and Income Problems | 0.011 | 43 | 0.010 | 38 |
| Cancer, Breast | 0.009 | 56 | 0.010 | 39 |

| Depression Screening | 0.008 | 60 | 0.010 | 40 |
| --- | --- | --- | --- | --- |
| History of Fracture | 0.008 | 61 | 0.010 | 41 |
| Marital Problem | 0.007 | 63 | 0.010 | 42 |
| Anxiety | 0.011 | 42 | 0.009 | 43 |
| Swallowing Evaluation and Treatment | 0.006 | 70 | 0.009 | 44 |
| Opioid Use Disorder | 0.006 | 71 | 0.009 | 45 |
| Urinary Incontinence | 0.002 | 84 | 0.009 | 46 |
| Original Medicare Entitlement | 0.015 | 14 | 0.008 | 47 |
| Tobacco Use | 0.012 | 32 | 0.008 | 48 |
| Asthma | 0.010 | 48 | 0.008 | 49 |
| Bipolar Disorders | 0.010 | 52 | 0.008 | 50 |
| Social Environmental Problems | 0.010 | 53 | 0.008 | 51 |
| Brain Injury | 0.007 | 65 | 0.008 | 52 |
| Cancer, Lung | 0.007 | 68 | 0.008 | 53 |
| Pelvic/Breast Exam | 0.005 | 72 | 0.008 | 54 |
| Muscular Dystrophy | 0.003 | 81 | 0.008 | 55 |
| Hypothyroidism | 0.013 | 24 | 0.007 | 56 |
| Hearing Impairment | 0.012 | 37 | 0.007 | 57 |
| Peripheral Vascular Disease | 0.011 | 39 | 0.007 | 58 |
| Fecal Incontinence | 0.007 | 64 | 0.007 | 59 |
| Drug Use Disorders | 0.015 | 17 | 0.006 | 60 |
| Diabetes | 0.012 | 34 | 0.006 | 61 |
| Primary Support Challenges | 0.011 | 45 | 0.006 | 62 |
| Access to Care Issues | 0.009 | 55 | 0.006 | 63 |
| Digital Rectal Exam | 0.003 | 82 | 0.006 | 64 |
| Human Immunodeficiency Virus  Screening | 0.001 | 88 | 0.006 | 65 |
| Human Immunodeficiency Virus and/or  Acquired Immunodeficiency Syndrome | 0.001 | 89 | 0.006 | 66 |
| Urinary Tract Infection | 0.019 | 5 | 0.005 | 67 |
| Obesity | 0.014 | 18 | 0.005 | 68 |
| Skin | 0.014 | 22 | 0.005 | 69 |
| Hyperlipidemia | 0.013 | 26 | 0.005 | 70 |
| Learning Disabilities | 0.011 | 47 | 0.005 | 71 |
| Spinal Cord Injury | 0.009 | 54 | 0.005 | 72 |
| Hip Fracture | 0.004 | 78 | 0.005 | 73 |
| Multiple Sclerosis | 0.003 | 83 | 0.005 | 74 |
| Illiteracy | 0.000 | 92 | 0.005 | 75 |
| Chronic Obstructive Pulmonary Disease | 0.013 | 23 | 0.004 | 76 |
| Family Conflict | 0.006 | 69 | 0.004 | 77 |
| Sex | 0.018 | 6 | 0.003 | 78 |
| Liver Disease | 0.013 | 25 | 0.003 | 79 |
| Intellectual Disabilities | 0.002 | 85 | 0.003 | 80 |
| X-ray | 0.010 | 50 | 0.002 | 81 |
| Suicidal Ideation | 0.010 | 51 | 0.002 | 82 |

| Mobility Impairment | 0.008 | 59 | 0.002 | 83 |
| --- | --- | --- | --- | --- |
| Migraine | 0.007 | 66 | 0.002 | 84 |
| History of Traumatic Brain Injury | 0.005 | 75 | 0.002 | 85 |
| Visual Impairment | 0.000 | 90 | 0.002 | 86 |
| Head/Neck Computed Tomography Scan | 0.012 | 36 | 0.001 | 87 |
| Other Developmental Delays | 0.005 | 73 | 0.001 | 88 |
| History of Physical Abuse and Rape | 0.004 | 76 | 0.001 | 89 |
| Cancer Endometrial | 0.002 | 86 | 0.001 | 90 |
| Spina Bifida | 0.001 | 87 | 0.000 | 91 |
| Cerebral Palsy | 0.000 | 91 | 0.000 | 92 |

**Note.** GB=gradient boosting; RF=random forest.

**Supplementary Table S12:** Model Performance Characteristics for Predicting EM

| EM -  Observed | | | | |  | | | | | |
| --- | --- | --- | --- | --- | --- | --- | --- | --- | --- | --- |
| Type | Cut  point Number |  |  |  |  |  |  | GINI  Impurity | F  Measure | Agree- ment |
|  |  | Prediction | Yes | No | AUC | Sen | Spec |  |  |  |
| Logistic  Regression - Full Model | 0.0009 | Yes | 643 | 300,673 | 0.7253 | 0.8007 | 0.4672 | 0.4976 | 0.0043 | 46.77% |
|  |  | No | 160 | 263,700 |  |  |  |  |  |  |
|  | | | | |  | | | | | |
| Logistic Regression - Selected  Model | 0.0009 |  |  |  |  |  |  |  |  |  |
|  |  | Yes | 643 | 304,731 | 0.7227 | 0.8007 | 0.4601 | 0.4966 | 0.0042 | 46.05% |
|  |  | No | 160 | 259,642 |  |  |  |  |  |  |
|  | | | | |  | | | | | |
| Random  Forest | 0.0010 | Yes | 695 | 324,957 | 0.7058 | 0.8119 | 0.4247 | 0.4874 | 0.0043 | 42.53% |
|  |  | No | 161 | 239,875 |  |  |  |  |  |  |
|  | | | | |  | | | | | |
| Gradient  Boosted Trees | 0.0240 | Yes | 690 | 325,163 | 0.6989 | 0.8061 | 0.4243 | 0.4873 | 0.0042 | 42.49% |
|  |  | No | 166 | 239,669 |  |  |  |  |  |  |
|  | | | | |  | | | | | |
| Multilayer  Perceptron Classifier | 0.0008 | Yes | 690 | 309,045 | 0.6995 | 0.8061 | 0.4529 | 0.4943 | 0.0044 | 45.34% |
|  |  | No | 166 | 255,787 |  | | | | | |

**Note**. AUC=Area Under the Curve; EM=Elder Mistreatment; GINI=Gini impurity; Sen=Sensitivity; Spec=Specificity.

**Supplementary Table S13:** ROC Curve Cut Points for the Logistic Regression and AI/ML Methods

| Model | Youden | Max Euclidean Distance | Concordance Probability | Index of Union | Sensitivity  >0.80 |
| --- | --- | --- | --- | --- | --- |
| Logistic Regression - Full Model | 0.001389 | 0.001389 | 0.001389 | 0.001389 | 0.00094 |
| Logistic Regression - Selected Model | 0.001521 | 0.001278 | 0.001278 | 0.001318 | 0.00094 |
| Random Forest | 0.001200 | 0.001250 | 0.001250 | 0.001200 | 0.00095 |
| Gradient Boosted Trees | 0.024230 | 0.024230 | 0.024230 | 0.024230 | 0.02401 |
| Multilayer Perceptron Classifier | 0.001190 | 0.001190 | 0.001190 | 0.001190 | 0.00080 |
| Logistic Regression - ADRD  Subgroup | 0.003825 | 0.00316 | 0.00316 | 0.00316 | 0.00243 |

**Supplementary Figure S1:** Receiver Operator Characteristic (ROC) Curve for Logistic Regression


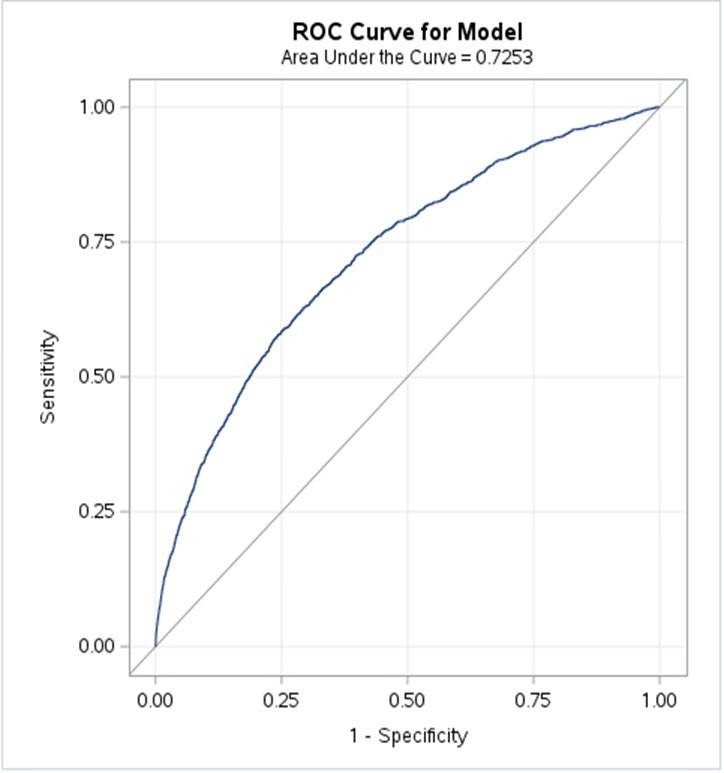


Using the Full Set of Variables

**Supplementary Figure S2:** Calibration Plots by Deciles of Observed Versus Predicted Probability of Elder Mistreatment (EM) Diagnosis Within 2 Years

Observed vs. Predicted EM Probability

0.006

0.005

0.004

0.003

0.002

0.001

0

1

2

3

4

5

6

7

8

9

10

Predicted Observed
